# The Effectiveness of Hard Insoles for Plantar Pressure in Cycling: A Crossover Study

**DOI:** 10.3390/bioengineering10070816

**Published:** 2023-07-08

**Authors:** Israel Casado-Hernández, Ricardo Becerro-de-Bengoa-Vallejo, Marta Elena Losa-Iglesias, Alfredo Soriano-Medrano, Daniel López-López, Emmanuel Navarro-Flores, Eduardo Pérez-Boal, Eva María Martínez-Jiménez

**Affiliations:** 1 Faculty of Nursing, Physiotherapy and Podiatry, University Complutense of Madrid, 28040 Madrid, Spain; isracasa@ucm.es (I.C.-H.); ribebeva@ucm.es (R.B.-d.-B.-V.); alfresor@ucm.es (A.S.-M.); evamam03@ucm.es (E.M.M.-J.); 2 Faculty of Health Sciences, University Rey Juan Carlos, 28933 Móstoles, Spain; marta.losa@urjc.es; 3 Research, Health, and Podiatry Group, Department of Health Sciences, Faculty of Nursing and Podiatry, Industrial Campus of Ferrol, Universidade da Coruña, 15403 Ferrol, Spain; daniellopez@udc.es; 4 Frailty Research Organized Group (FROG), Department of Nursing, Faculty of Nursing and Podiatry, University of Valencia, 46010 Valencia, Spain; 5 Faculty of Nursing and Physiotherapy, University of León, 24401 Ponferrada, Spain; epereb@unileon.es

**Keywords:** cycling, plantar pressure, foot insole, metatarsal head, in-shoe

## Abstract

Background: Hard insoles have been proposed to decrease plantar pressure and prevent foot pain and paresthesia due to repetitive loading. The aim of this research was to analyze the effect of three different hard insoles in cycling on healthy subjects. Methods: A crossover randomized trial was carried out. The mean age of the subjects was 35 ± 3.19 years, and all of them were men. While the subjects were cycling on a stationary bicycle, their plantar pressure was recorded with nine in-shoe sensors placed in nine specific foot areas to test a standard ethylene-vinyl-acetate 52° Shore A hardness insole, a polypropylene 58° Shore D insole, and a polypropylene 58^0^ Shore D insole with selective aluminum 60 HB Brinell hardness in the metatarsal head and hallux. Results: The maximum plantar pressure decreased significantly with the polypropylene insole containing selective aluminum in the metatarsal head and hallux areas. The maximum plantar data of the polypropylene aluminum insole in the M2 area (5.56 kgF/cm^2^), fifth metatarsal styloid process (6.48 kgF/cm^2^), M3–M4 area (4.97 kgF/cm^2^), and hallux (8.91 kgF/cm^2^) were of particular interest compared to the other insoles. Conclusions: The use of insoles made of polypropylene with aluminum in the metatarsal head and hallux areas decreases the maximum plantar pressure in cycling compared to standard EVA and polypropylene insoles.

## 1. Introduction

Cycling has great health benefits for those who practice it as well as for the environment. Cycling aims to reduce CO2 emissions by increasing quality of life (QoL) and the quality of the ecosystem [1]. Those who practice cycling indoors can improve their health and quality of life as well as decrease their lipid profile, blood pressure, and weight [2,3,4]. In order to achieve such benefits, governments have to implement safer cycling infrastructure [5].

In addition, cycling is good for quadricep muscle rehabilitation because one can control the stress on knee ligaments by modifying the position of the pedals and the height of the seat [6]. Subjects with knee osteoarthritis that perform stationary cycling exercises can decrease knee pain, but it is not clear that this exercise improves stiffness and QoL compared to other exercises [7]. Cyclists with bad knee alignments can develop injuries from overuse and pain related to the vastus medialis and vastus lateralis, altering the activation of the hamstring and quadricep muscles and increasing knee adduction and ankle dorsiflexion [8].

The biomechanical lower limb characteristics of cycling include longitudinal metatarsal arch and medial longitudinal arch collapse, plantar fascia stretching, subtalar joint pronation, internal tibial rotation, knee valgus, internal femur rotation, and eccentric contraction of the external muscle rotators of the hip [9,10].

The lower limb muscles involved in cycling have to adapt their function according to the mechanical demands of each pedaling phase. These muscles shift their contractile functions in different environmental and mechanical situations to improve pedaling performance and in rehabilitation therapies. Cycling with a faster cadence produces a phase advance in the crank top dead center and causes the muscles to contract like a spring [11].

The energy generated by the cyclist to move the bicycle is due to the relationship between three segments: the foot, the shoe, and the bicycle pedal. These three segments together produce a cyclic movement that drives the bicycle forward [12,13,14]. There is a close relationship between the loads and the repeated pedaling movement in cycling that causes an increase in the reaction forces between the foot and the pedal [15,16]. Sport cycling is recommended due to the lower impact rates produced at the articular joint level, despite the fact that there are half as many bodyweight forces when seated and three times as many when cycling in a standing position [17,18]. Due to the increase in forces transmitted by the lower limb to the feet on the pedals, high plantar pressures are generated, which can develop into injuries such as paresthesia, numbness of the forefoot owing to compression of the plantar nerves, metatarsalgia, and plantar muscle overuse [19,20].

Previous studies have analyzed plantar pressures during the seated cycle phase and revealed a consistent pattern in which the greatest plantar pressure is located in the forefoot [21,22]. Other research has indicated that the most overloaded forefoot areas are the I metatarsophalangeal joint and hallux [16,23]. An increased load on the foot causes a medial shift in forefoot pressure, demonstrating a greater reliance on the load-bearing anteromedial structures of the forefoot [16].

Wearing insoles inside cycling shoes produces plantar pressure redistribution. According to the insole material manufactured, the plantar pressure distribution can oscillate between decreasing or increasing the pressure [24,25]. On the other hand, contoured insoles modify the contact area and the plantar pressure distribution in cycling, thus increasing the pressure in the hallux area compared to flat insoles with no perceived differences in comfort [26]. A previous study assessed the highest forefoot plantar pressure variations in cyclists by contrasting a carbon fiber insole with a plastic insole. The results showed an 18% increase in the forefoot area using the carbon fiber insole compared to the plastic insoles, which were less stiff [21].

An in-shoe plantar pressure system is a reliable tool to measure and analyze plantar loading variations occurring in any kind of sport or walking [27,28]. According to the results in cycling, the in-shoe system allows analysis of the plantar pressure in different plantar foot areas and to avoid injuries caused by incorrect biomechanical movements [20]. Maximum plantar loading in cycling is in the forefoot. Biomechanical performance and the influence of the parts in the forefoot area can produce injuries such as ischemia and metatarsalgia because of the increase in peak plantar pressure [21].

Cycling practice can reduce plantar pressure compared to other cardiovascular exercises and is a good sport for those with forefoot diseases and subjects who suffer from metabolic diseases that involve any functional recovery [29]. The aim of this research was to analyze the plantar pressure variations in cyclists using insoles of varying hardness in specific foot areas. Our hypothesis is that insoles with aluminum in the forefoot and hallux areas decrease plantar pressure.

## 2. Materials and Methods

### 2.1. Study Design

A crossover randomized clinical trial was performed between October and December 2022, and the effect of different foot insoles manufactured with different hardness in the forefoot and hallux was evaluated to analyze the plantar pressure variability. This research was authorized by the ethics committee at the Complutense University of Madrid (consent nº CE_20221013-15_SAL). All the subjects signed an informed consent form prior to participation.

All of the subjects recruited for the research were men, and all of them were recreational cyclists who ride on weekends. The mean age of the subjects were 35 ± 3.19 years old with an age range between 28 and 39 years old. Their sociodemographic characteristics are shown in Table 1.

Table 1 shows the sociodemographic characteristics of the eighteen healthy cyclists who took part in the study.

The subjects were previously screened by an expert podiatrist in the biomechanics of cycling with 10 years’ experience. The inclusion criteria were: (1) subjects older than 18 years old and younger than 45 years old, (2) healthy cyclists without musculoskeletal or neurological diseases that could influence the lower extremities at the time of testing, (3) participants without lower limb asymmetries, and (4) cyclists who signed and understood the informed consent document. The exclusion criteria included (1) subjects younger than 18 years old and older than 45 years old, (2) with musculoskeletal disorders, (3) with lower limb asymmetry, (4) with cardiovascular disturbance, or (5) who refused to sign the informed consent document.

The sample size was calculated with software from the Clinical and Biostatistical Epidemiology Unity, University Hospital Complex of La Coruña, University of A Coruña (www.fisterra.com (accessed on 01 September 2022)). We set out testing differences in maximum plantar pressures based on other research employing ethylene vinyl acetate (EVA) material. The maximum metatarsal pressure observed in cyclists using a custom flat insole (CFI) and a contoured insole was 148.4 ± 35.2 and 147.2 ± 29.8 KPa, respectively [26]. To accomplish this, a statistical confidence of 95%, with a two-tailed hypothesis test and a large effect size of 0.90, an α-error of 0.05, and a power of analysis of 0.80 (β error = 20%) were required [30]. The minimum sample size was eight participants.

The research was accomplished following the guidelines and list for the Template for Intervention Description and Replication (TIDieR) [31].

### 2.2. Method

The research was carried out using three different flat insoles with different hardness levels. One insole made of EVA of 3 mm thickness and 52° Shore A hardness (CONTROL), another insole made of polypropylene (58° Shore D) of 2 mm and an EVA lining 52° Shore A hardness of 1 mm thickness with a total thickness of 3 mm (POLI), and the final insole made of polypropylene (58° Shore D) of 2 mm with a fenestration in the metatarsal heads area and hallux filling with aluminum (60 HB Brinell hardness) of 2 mm and an EVA lining 52° Shore A hardness of 1 mm thickness with a total thickness of 3 mm (ALUM). The hardness of the insole was measured with a durometer (durometer model Vickers PCE-1000, PCE Ibérica S.L. Tobarra, Albacete, Spain). Each subject wore their own shoes to perform the test and cotton socks with a plain knitted structure. The testing order with each insole inside the shoe was randomized.

A wireless embedded sensor system was used to analyze the in-shoe pressure, with the W-INSHOE (https://www.medicapteurs.com/produits/winshoe-2/ (accessed on September 2022)) consisting of two ultra-lightweight units (50 g), each controlling nine ultra-thin, calibrated resistive sensors [32]. The sensors were positioned on the insoles in specific foot areas and recorded the pressure of target plantar zones in real time via Bluetooth. The measured data of the maximum plantar pressure were transmitted via Bluetooth at 100 Hz to a laptop. The sensors were calibrated prior to data acquisition. The sensors were fixed to the insoles in nine foot areas: (1) the hallux area (HALLUX), (2) the first metatarsal head area (M1), (3) the second metatarsal head area (M2), (4) the third and four metatarsal head areas (M3-M4), (5) the fifth metatarsal head area (M5), (6) the fifth metatarsal styloid process, (7) the calcaneus, (8) the medial plantar calcaneus, and (9) the lateral plantar calcaneus. Once the sensors were placed on the insole, the insole was placed in the cyclist’s shoe directly beneath the sock-covered foot.

The data capture software was F-PRINT Software V 1.247^®^. The software divided the plantar surface into nine areas as previously described, according to the sensor foot area placement. Previously, age, weight, height, and foot size were recorded. The cycling trials were carried out by the subjects using a static bike. The bike and the seat position were arranged according to the subject’s anthropometry. The crank length and frame size were the same as on the participants’ own bikes. The position for bike riding was standardized with the participants’ hands on the drop bars. They were instructed to remain seated during each ride. The subjects wore their own conventional cycling shoes which were attached to the pedals with straps and toeclips during the test. The cadence was tested with a cycle ergometer at 90 rpm (rotations per minute) and the power output (workload) was 200 W (Watts). Prior to data acquisition, every subject was given a briefing warm-up and rode free of power outputs and pedaling rates for a 10-min duration. Once the warm-up was over, randomized insoles testing was performed, and data acquisition was conducted during the last 30 s after pedaling freely for 5 min. The participants rested between testing the insoles for at least 4 min to assure appropriate recovery.

### 2.3. Statistical Analysis

All data were analyzed for normality values according to the Shapiro–Wilk test, and the data were tested regarding normal distribution if *p* > 0.05. To describe the cyclists‘ sociodemographic characteristics, the variables recorded were the mean, median, body mass index (BMI), standard deviation (SD), and 95% confidence interval (95% CI). We used the nonparametric Wilcoxon test to assess non-statistically significant differences among the CFI groups. Statistically significant differences in maximum pressures between the CFI groups were measured using an independent *t* test.

A *p*-value < 0.05 with a confidence interval of 95% was considered statistically significant for all tests (SPSS, v 20.0; SPSS inc., Chicago, IL, USA).

## 3. Results

The mean of the maximum plantar pressure is shown in Table 2. The maximum plantar values were located predominantly in the forefoot area, highlighting the second metatarsal head (M2) (588.22 kgF/cm^2^), followed by the fifth metatarsal styloid process (342.20 kgF/cm^2^), the third and fourth metatarsal heads (M3–M4) (247.21 kgF/cm^2^), and the hallux (131.93 kgF/cm^2^) for each different insole. The effect of each custom flat insole is statistically significant in the interaction of the maximum plantar pressure data, resulting in a decrease with the harder the material analyzed. The values decreased significantly in all plantar foot areas using harder insoles. The maximum plantar data using the polypropylene aluminum insole in the M2 area (5.56 kgF/cm^2^), the fifth metatarsal styloid process (6.48 kgF/cm^2^), the M3-M4 area (4.97 kgF/cm^2^), and the hallux (8.91 kgF/cm^2^) were especially noteworthy.

In Table 3, a reduction in maximum forefoot pressure established a great effect size when the polypropylene and aluminum insole was used compared with the EVA control standard. All of the variables showed statistically significant values when the plantar pressure decreased with a harder insole (*p* < 0.05) except in the lateral plantar calcaneus with a *p* = 0.271.

## 4. Discussion

The purpose of this research was to analyze plantar pressure variations when wearing harder flat insoles in the cyclist’s own shoes and compare the results between a control standard EVA insole and a polypropylene insole and a polypropylene insole with aluminum in the metatarsal and hallux areas. As a novelty, this is the first research to investigate plantar pressure variation with an aluminum insole in cyclists. When comparing foot flat insoles inserted into cyclists’ shoes, a variation in maximum plantar pressure was shown, decreasing when using the harder insole in comparison with a standard EVA insole.

Baur et al. [25] compared the plantar pressure distribution in cyclists using a standard insole and a carbon fiber insole inside the cyclists’ shoes and concluded that there was a peak plantar pressure decrease in the rearfoot and central and first metatarsal head areas but an increase in toe regions and the fifth metatarsal area. Regarding our research, all of the forefoot plantar pressure values decreased using the aluminum insoles, and the peak plantar pressure was 9.24 ± 0.89 kgF/cm^2^ in the fifth metatarsal head followed by 8.77 ± 0.28 kgF/cm^2^ in the hallux.

Jarboe et al. [21] analyzed plantar pressure distribution using a carbon-fiber-made cycling shoe and a manufactured cycling shoe with plastic soles concluding that the use of carbon fiber cycling shoes increased the peak plantar pressure by 18% with the use of carbon fiber. According to our research the combination of the use of harder flat insoles with standard cycling shoes significantly reduced the maximum plantar pressure in all of the foot areas, avoiding aggravating metatarsalgia and ischemic foot conditions in cyclists.

Based on our research, the plantar pressure of the fifth metatarsal styloid process decreased during cycling when using harder insoles, the peak pressure with the standard EVA insole was 342.20 kgF/cm^2^, with the polypropylene insole, it was 14.48 kgF/cm^2^, and with the polypropylene and aluminum insole, it was 6.48 kgF/cm^2^. These data were statistically significant in avoiding or preventing injuries in the fifth metatarsal, as opposed to the research of Queen et al. [33], who analyzed the effect of a rigid carbon graphite footplate on plantar loading of the fifth metatarsal performing two agility tasks, concluding that the carbon graphite footplate modified the plantar loading but not enough to avoid or decrease the risk of fifth metatarsal stress fracture.

Moreover, the research conducted by Bousie et al. [24] evaluated the variation of plantar pressure in cycling using hard and soft insoles with a medial or lateral forefoot post and concluded that soft insoles increased the plantar pressure without affecting comfort and the forefoot post modified the plantar pressure, affecting comfort. In accordance with our research, flat insoles were used, and the hardest one’s data showed the lowest plantar pressures in all foot areas, i.e., the polypropylene with aluminum in the metatarsal head insoles were the ones which most decreased the plantar load.

According to the research performed by Sanderson et al. [34], whereby they analyzed the influence of the cadence in competitive and recreational cyclists on plantar pressure, the authors concluded that the primary load joints were the first metatarsal and the hallux. According to our research, the fifth metatarsal head and the hallux suffered the highest maximum plantar load using the polypropylene with aluminum in the metatarsal heads insole compared with the other insoles. In the same study, pedal force application did not show a specific plantar pressure influence.

On the other hand, Yeo et al. [35] analyzed the effect of foot orthosis on the crank power output in a stationary cycle ergometer at maximum power and concluded that the use of custom-made orthoses did not affect the power capacities and torque cycling at maximal power. In addition, Sanderson et al. [16] analyzed the cadence and power output influence between competitive and recreational cyclists, and according to their results, power outputs of up to 235 W did not show differences between the groups in force application during steady cycling, and regarding cadence, these authors analyzed three different cadences, at 100 rpm, 80 rpm and 60 rpm among the groups, and concluded that the highest cadence at 100 rpm decreased the force application. According to our research, all the subjects performed the protocol at the same cadence of 90 rpm and a power output of 200 W, a normal situation that did not influence the final effect of the plantar pressure, but the plantar pressure was different with the different hardness of the insoles.

Casado et al. [36,37] analyzed the variation in the plantar pressure with hardness using noncontoured insoles made with polypropylene and aluminum in the metatarsal head and EVA 52° Shore A in motorcycle sport and concluded that the plantar pressure generated by the rider on a motorcycling simulator decreased using hard insoles. Taking into consideration the similarity in the insoles used, in our research, the plantar pressure was preceded by a cycling movement of foot–shoe–pedal, in contrast to the motorcycle that only used a support movement, but nevertheless, in both situations using similar insoles, in all cases, the plantar pressure decreased.

Previous research by Henning et al. [23] analyzed plantar pressure in cycling using two different shoe types, running shoes and conventional cycling shoes, and concluded that the use of a shoe with a soft insole, like running shoes, produces an increase in the load in the metatarsal areas, improving the plantar pressure and injury risk, but the principal peak plantar pressures occurred in the hallux which redistributed this pressure in the metatarsal area; nevertheless, the conventional cycling shoes with a hard insole distributed the load around the foot, decreasing the peak plantar pressure. According to our research results, the peak plantar pressure using the polypropylene insole with aluminum in the metatarsal head and the hallux was 9.24 ± 0.89 kgF/cm^2^ in the fifth metatarsal head followed by 8.77 ± 0.28 kgF/cm^2^ in the hallux compared to using the EVA insole with the peak plantar pressure of 98.81 ± 15.30 kgF/cm^2^ in the fifth metatarsal head using the polypropylene insole with aluminum in the metatarsal head and hallux, and 122.14 ± 19.68 kgF/cm^2^ in the hallux.

A major limitation of our research is that the trial testing period was comparatively short in comparison to the period spent cycling in a typical competition or during training. The results of the research surely imply that using harder insoles and with aluminum in the metatarsal head area and hallux decreases cycling pressure. In this way, it would be interesting to analyze longer periods of cycling time with increased training intensity in futures studies. Another limitation is the use of the same cadence and workload cycling for all the participants because the aim of our research was to compare the hardness of different insoles. Future research analyzing and comparing the differences in peak plantar pressure with different cadences and power using different insoles would be interesting. Furthermore, for future investigations, research on the effect of the hardness of insoles and the relationship with the ankle joint force generated could be interesting to investigate. Another limitation of our research was the lack of influence of the inertial forces of the ground in cycling as well as climbing hills and the effect on the plantar pressure with different insole hardness values, and finally, the influence of using different manufactured shoes in the variation of plantar pressure could be important in clinical assessment.

## 5. Conclusions

The use of insoles made of polypropylene with aluminum in the metatarsal head areas and the hallux versus standard EVA and polypropylene insoles decreases the maximum plantar pressure in sport cycling. In addition, the reduction in the maximum forefoot pressure may help to decrease any overload-related issues that could affect cyclists’ bones, muscles, ligaments, or soft tissue.

As a final point, wearing harder insoles when cycling reduces plantar pressure and increases QoL and cycling performance.

## Figures and Tables

**Table 1:** Anthropometric and sociodemographic characteristics.

Variables	Mean ± SD (*n* = 18)	Range (Min–Max) (*n* = 18)	95% CI (*n* = 18)
Age (years)	35 ± 3.19	28–39	33.52–36.70
Height (cm)	177 ± 0.05	164–180	172.42–177.80
Weight (kg)	73.64 ± 5.72	62.40–81.80	70.79–76.49
BMI (kg/m^2^)	23.99 ± 1.08	21.97–25.31	23.10–24.90
Foot Size	42.22 ± 2.86	37–47	40.02–44.42

**Abbreviations:** SD: standard deviation; Min: minimum; Max: maximum; 95% CI: 95% confidence interval; cm: centimeters; kg: kilograms; BMI: body mass index; kg/m^2^: kilograms/meter^2^.

**Table 2:** Maximum plantar pressure areas measured with in-shoe pressure sensors used in right and left foot cycling with different insole hardness values and the comparisons effect.

Variables (kgF/cm^2^)	Control Standard EVA		Polypropylene		Polypropylene Aluminum	
	Mean ± SD (95% CI)	Median (95% CI)	Mean ± SD (95% CI)	Median (95% CI)	Mean ± SD (95% CI)	Median (95% CI)
Lateral Plantar Calcaneus	10.40 ± 0.31 (10.25 to 10.56)	10.66 (10.00 to 10.66)	10.12 ± 0.88 (9.68 to 10.57)	10.33 (9.33 to 10.86)	6.16 ± 0.40 (5.96 to 6.36)	6.00 (6.00 to 6.33)
Medial Plantar Calcaneus	4.72 ± 1.05 (4.19 to 5.24)	5.00 (4.00 to 5.53)	1.18 ± 0.26 (1.05 to 1.31)	1.00 (1.00 to 1.33)	1.42 ± 0.31 (1.26 to 1.58)	1.33 (1.33 to 1.66)
Calcaneus	4.35 ± 1.05 (3.82 to 4.87)	4.16 (3.66 to 5.00)	1.12 ± 0.25 (1.00 to 1.25)	1.00 (1.00 to 1.33)	1.22 ± 0.32 (1.06 to 1.38)	1.33 (1.00 to 1.33)
Fifth Metatarsal Styloid Process	324 ± 35.94 (306.45 to 342.20)	313.66 (305.46 to 333.49)	14.31 ± 0.35 (14.13 to 14.48)	14.33 (14.00 to 14.53)	6.31 ± 0.35 (6.13 to 6.48)	6.33 (6.00 to 6.53)
M5	98.81 ± 15.30 (91.20 to 106.42)	97.83 (90.26 to 107.00)	11.61 ± 0.47 (11.37 to 11.84)	11.66 (11.33 to 12.00)	9.24 ± 0.89 (8.79 to 9.68)	9.33 (8.46 to 10.00)
M3-M4	240.77 ± 12.94 (234.34 to 247.21)	241.50 (231.93 to 251.40)	6.25 ± 0.76 (5.87 to 6.63)	6.00 (5.66 to 6.86)	4.77 ± 0.39 (4.58 to 4.97)	4.83 (4.46 to 5.00)
M2	584.07 ± 8.34 (579.92 to 588.22)	587.16 (579.11 to 589.86)	15.57 ± 0.89 (15.13 to 16.01)	15.50 (14.79 to 16.33)	5.37 ± 0.39 (5.17 to 5.56)	5.33 (5.00 to 5.66)
M1	88.64 ± 12.54 (82.40 to 94.88)	88.33 (78.73 to 95.40)	1.22 ± 0.30 (1.07 to 1.37)	1.16 (1.00 to 1.33)	1.22 ± 0.32 (1.06 to 1.38)	1.33 (1.00 to 1.33)
Hallux	122.14 ± 19.68 (112.35 to 131.93)	125.66 (108.98 to 134.93)	10.92 ± 0.35 (10.75 to 11.10)	11.00 (11.00 to 11.00)	8.77 ± 0.28 (8.63 to 8.91)	8.66 (8.66 to 9.00)

**Abbreviations:** kgF: kilogram force; cm^2^: square centimeters; EVA: ethylene vinyl acetate insoles; SD: standard deviation; 95% CI: 95% confidence interval; M1: first metatarsal area; M2; second metatarsal area; M3; third metatarsal area; M4: fourth metatarsal area; M5: fifth metatarsal area.

**Table 3:** Comparison between the customized foot insoles on the effect of the plantar pressures of the cyclists.

Variables (kgF/cm^2^)	Control vs. Poly *p*-Value	Control vs. Alum *p*-Value
Lateral Plantar Calcaneus	0.271	0.001
Medial Plantar Calcaneus	0.001	0.001
Calcaneus	0.001	0.001
Fifth Metatarsal Styloid Process	0.001	0.001
M5	0.001	0.001
M3-M4	0.001	0.001
M2	0.001	0.001
M1	0.001	0.001
Hallux	0.001	0.001

**Abbreviations:** kgF: kilogram force; cm^2^: square centimeters; Control: control standard EVA insole; Poly: polypropylene insole; Alum: polypropylene and aluminum in forefoot and hallux areas insole; M1: first metatarsal area; M2; second metatarsal area; M3; third metatarsal area; M4: fourth metatarsal area; M5: fifth metatarsal area. *p*-value < 0.05 with a confidence interval of 95% was considered statistically significant.

## Data Availability

The dataset supporting the conclusions of this article is available from isracasa@ucm.es (Faculty of Nursing, Physiotherapy and Podiatry Complutense University of Madrid) upon request.
